# Infants in Control—Evidence for Agency in 6‐ to 10‐Months‐Old Infants in a Gaze‐Contingent Eye Tracking Paradigm

**DOI:** 10.1111/cdev.70022

**Published:** 2025-07-25

**Authors:** Florian Markus Bednarski, Katrin Rothmaler, Simon M. Hofmann, Charlotte Grosse Wiesmann

**Affiliations:** ^1^ Research Group Milestones of Early Cognitive Development Max‐Planck‐Institute for Human Cognitive and Brain Sciences Leipzig Germany; ^2^ Department of Philosophy Leipzig University Leipzig Germany; ^3^ School of Psychology The University of Auckland Auckland New Zealand; ^4^ Department of Neurology Max Planck Institute for Human Cognitive and Brain Sciences Leipzig Germany; ^5^ Cognitive Neuroscience Lab, Department of Liberal Arts and Sciences University of Technology Nuremberg Nuremberg Germany

**Keywords:** agency, agentic control, contingency paradigm, control, eye tracking, gaze‐contingent, infancy

## Abstract

The ability to control movement is a core element of agency. Previous studies of infant agency have focused on responses to sensory contingencies but neglected the importance of infants' control as a necessary indicator of agency. Here, we test whether infants flexibly control their eye movements with a gaze‐contingent eye tracking paradigm. Infants aged 6–10 months (*N* = 45, 18 female, recruited in a city of about 600.000 inhabitants in Germany in 2022) were presented images hidden under a unicolored surface, which they could scratch free by gazing over the screen. Results show that infants flexibly directed their gaze to areas with most information in the underlying image. This indicates that infants can flexibly adjust their gaze to changing circumstances.

From very early in life, infants vividly respond to stimulation with bodily motions. For example, when infants are presented with a jiggling overhead mobile, they often smile and start to kick with their limbs (Rovee and Rovee 1969). However, differentiating bodily movements in response to stimulation from *actions* that are initiated and controlled by the infant is a difficult task. Previous research has employed contingency paradigms to test infants' abilities to respond to sensory stimuli (Rochat and Striano 2000; Rovee‐Collier et al. 1978; Zmyj et al. 2011). This approach has been criticized for methodological imprecision (Zaadnoordijk et al. 2018), lacking standardization (Sen and Gredebäck 2021) and missing an indicator of agentic control, a critical component of agency (Bednarski et al. 2022). In this paper, we report results from a novel gaze‐contingent eye tracking paradigm designed to answer the following research question: Do infants flexibly control their eye movements, and how does this control develop within the first year of life?

When evaluating agency, it is important to differentiate bodily movements that are controlled by an agent from those movements that are merely happening to them (Bednarski et al. 2022; Frankfurt 1978). Imagine two instances of an infant kicking with their leg. In the first instance, a doctor is testing the infant's patellar reflex, and the leg movement is merely happening as an automatic response to the hit below the infant's knee. In the second instance, the infant decides to kick their leg because they do not want to be hit by the doctor. From an observer's perspective, both movements might look exactly alike; thus, for this observer, it is impossible to know whether the infant is in control over the motion—qualifying it as an action—or if the motion is caused by a reflex. Differentiating these two kinds of bodily movement requires a measure of agentic control.

A characteristic of agentic control is that an agent has to be able to adapt their behavior to new circumstances. It has therefore been argued that agentic control can be measured by manipulating external circumstances, under which a behavior is displayed, and testing whether the response behavior is flexibly adapted to these changing circumstances (Bednarski et al. 2022). A flexible response to the change in circumstances would reveal agentic control by the agent. An experimental paradigm testing for infant agency therefore needs to show that bodily motion of an infant is not just a response to rewarding stimuli, but that the infant flexibly controls their movements and is able to adapt them to the circumstances. Note that the present study tests whether infants possess agentic control and not whether they perceive themselves as agents (i.e., have a sense of agency).

Previous studies investigating the development of agency in the first 12 months of life have primarily used sensory contingency paradigms (for a review see e.g., Bednarski et al. 2022). Contingency paradigms utilize infants' early abilities to learn behaviors via reinforcement (Jacquey et al. 2020; Rochat and Striano 2000; Rovee‐Collier et al. 1978; Sen and Gredebäck 2021). A classic example of a contingency paradigm is the mobile paradigm (Rovee and Rovee 1969). In this paradigm, contingency between an infant's leg motion and an overhead mobile is created by connecting the two with a ribbon. Across several studies (Lewis et al. 1990; Rovee and Rovee 1969; Rovee‐Collier and Gekoski 1979), a robust increase in kicking frequency during the contingency and right after disconnecting the leg from the mobile was observed. This was interpreted as evidence that infants control the mobile with their leg motion (Rochat and Striano 2000). However, the increase in kicking frequency in the contingent phase can also be explained by reinforcement learning that does not necessarily entail flexible control over this behavior. Some researchers argued that increased leg movements after disconnecting the mobile showed that infants had understood themselves as causal agents and were probing the contingency (Rochat and Striano 2000). However, this behavior as well lacked an index of control by the infant (Bednarski et al. 2022). In particular, the increased kicking was not found to be specific to the leg that had previously been connected to the mobile (Watanabe and Taga 2006), suggesting that infants did not probe the contingency in a controlled way. Instead, increased kicking may have been an uncontrolled emotional expression, such as frustration, about the sudden absence of the rewarding stimulus (Bednarski et al. 2022).

In sum, contingency paradigms, like the mobile paradigm, have produced evidence that infants from very early in life are able to learn multisensory contingencies, which affect their movements. However, these studies do not allow conclusions about infants' control over their bodily movement and thus cannot answer the question of what extent infants are agents.

To test the development of infants' motor control, a different line of research investigated infants' reaching and grasping abilities, and the extent to which they adapt these motions to changing difficulty levels (Adolph and Robinson 2015; Gottwald 2018; von Hofsten 2004). For example, Gottwald (2018) measured infants' arm velocity when picking up an object and moving it to a container. By changing the distances to and size of the container, they showed that 14‐month‐olds adjust the velocity of their motion depending on the task difficulty. To encourage infants to perform the grasping movements, their caregiver demonstrated the exact same movement to them multiple times. These findings leave open the question to what extent infants can flexibly adapt and control actions, beyond movements anchored in their motor repertoire (such as grasping) and, independently of imitating another agent.

Furthermore, infants in the above studies on motor control were 14 months or older. Indeed, the development of reaching and grasping is protracted compared to other modalities, like vision. While infants first begin to reach for and grasp stationary objects between 4 and 6 months (Adolph and Robinson 2015; von Hofsten 1989), these movements remain fairly clumsy until their second year of life when infants begin to prospectively control their limb movements (Gottwald et al. 2016). This protracted development of prospective limb motion control is preceded by prospective shifts of eye movements which already emerge by 3–4 months of age (Canfield and Kirkham 2001). By 9–10 months of age, infants show developing degrees of inhibitory control over their eye movements (Oakes et al. 2002). Therefore, to study the earliest development of agentic control, a shift from gross motor development to an earlier developing modality such as eye movement is warranted.

Several experimental designs have used eye tracking to establish contingencies between infants' gaze and the presentation of visual stimuli on a screen (Kanakogi et al. 2022; Miyazaki et al. 2014; Wang et al. 2012). These studies show that infants can learn contingencies between their gaze and visual stimuli and use these to produce effects on the screen (Kanakogi et al. 2022). Specifically, in Kanakogi et al. (2022), 8‐month‐old infants directed their gaze to an aggressive agent if they had previously seen that this caused the agent to be punished. This indicates that infants from at least 8 months can direct their gaze to visible targets to produce effects on the screen. In the context of flexible agentic control, these findings raise the question of whether infants can also control their gaze to flexible locations without reacting to a visible target associated with a specific effect. In Miyazaki et al. (2014), infants were presented with a black screen, but as soon as they directed their gaze to the screen, a small circle of an underlying colorful picture stimulus was uncovered at the location of the infant's gaze point. This way, infants could gradually scratch free the underlying image with their gaze. The authors found that 8.5‐month‐old infants looked as much to the black areas of the screen as adults did who reported having detected and tried to control the contingency. They interpreted this as an indication that infants as well may have understood the contingency and tried to scratch free the screen. However, similar to the traditional mobile paradigm, the recorded looking behavior lacked an index of infants' flexible control over the contingency. Specifically, infants were able to scratch free the entire screen without needing to direct their gaze to specific locations. This was the same across all trials without the need to flexibly adapt their gaze depending on the affordance of the trial.

In the present study, we built on this paradigm and extended it by introducing specific, changing target locations in the underlying images and a measure of infants' specific adaptation to these changing circumstances. Our goal was to test (1) infants' capacity to flexibly adapt their eye movements to changing circumstances, and (2) how this marker of agentic control develops between 6 and 10 months of age. The central idea was to manipulate the contingency to test how infants respond to a change in the circumstances under which they learned about the contingent relation between their gaze and the stimulus. In particular, we derived two critical aspects for measuring flexible control in the above setting. First, a systematic variation of circumstances that predict systematic changes in behavior as a specific measure of control. Second, building on criticisms of the mobile paradigm (see e.g., Bednarski et al. 2022), we tested whether infants would show a specific controlled response to a disruption of the contingency.

Specifically, the underlying images varied systematically with respect to where information could be found. Infants saw these images before they were covered and could be scratched free. This way, we created specific expectations of where interesting content could be revealed, allowing us to test whether infants specifically gazed at these areas and flexibly adapted their gaze behavior depending on where the content could be revealed. This novel design allowed us to measure infants' control over their gaze based on two behavioral signatures. In the contingent phase, we expected infants to direct their gaze to screen locations where they could expect to reveal more informative content. This resulted in a specific measure of control in contrast to the previously used overall increase in response to the contingency. Second, we introduced a disruption of the contingency. That is, after a while infants could no longer scratch free further parts of the image, but instead, a still image of the last result of their gaze scratching was displayed. In this phase, we expected that infants would direct their gaze to yet‐to‐be‐revealed areas of the image, again systematically gazing at those areas with more informative underlying contents. If confirmed, we reasoned that this would show that infants learned the contingency, remembered the location of informative visual input, and critically, were able to direct their gaze in a flexible way to where they expected to be able to reveal information. Thus, manipulating where information could be expected made specific predictions about where exactly infants would look on the unicolored screen, and thus provided us with a measure of flexible control both in the contingent and in the disruption phase.

## 1 Methods

This study was preregistered at https://aspredicted.org/kvjs‐bxv5.pdf. All analyses were conducted according to this preregistration unless explicitly stated otherwise.

### 1.1 Participants

*N* = 52 infants participated in our study, of which 7 had to be excluded because they did not contribute at least two trials for each image condition (drop/rise). Thus, *N* = 45 infants were included in the analysis (18 female, 27 male, mean age = 261 days, median = 259, SD = 42.67). Overall, these 45 infants contributed 1955 recorded trials; 465 of these were excluded (exclusion rate of 24%, cf. Data S1) according to criteria stated in the preregistration (i.e., insufficient looking time to the screen). Participants were recruited via mail to sign up for a database of families located in a city of about 600.000 inhabitants. Parents gave informed consent for the participation of their infants. Infants could choose a small gift after participating, and parents were compensated monetarily for their travel expenses. The experiment was approved by the university ethics committee.

### 1.2 Materials

**FIGURE 1:**
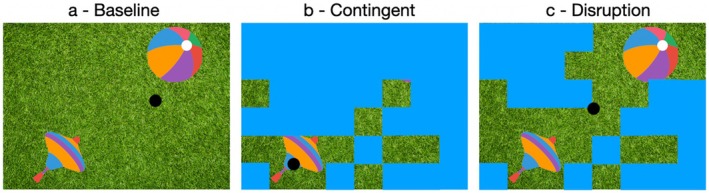
Illustration of a gaze scratch paradigm trial. From left to right, baseline, contingent and disruption phase. The baseline phase (a) lasted 5 s, in which infants see the image that will be covered by the blue surface in the next phase and can thus see the location of two objects on this image. The transition between the baseline and contingent phase, lasted 3 s and intended to disengage the infants from the object locations while at the same time showing that the image was being covered with a unicolored cover. In the contingent phase (b), infants were able to uncover the image with their gaze until either 30 s had passed or until 20% of the cover had been removed. The disruption phase (c) lasts 5 s, in which the screen no longer changes in reaction to the infants' gaze and they see the still image that resulted from their previous scratching action.

Infants were presented with images of two salient objects (toys, animals, figures etc.) on a uniform background (grass, sand, sky etc.). The objects were placed in opposite corners of the image. There were two conditions: In the image condition rise, the objects were located in the bottom left and top right corners of the image. If an image belonged to the condition drop, the objects were located in the top left and bottom right corners of the image. In total, 32 images were created from 16 object pairs and 16 background images so that each object pair occurred once for each condition (i.e., drop and rise). The stimuli set was split into two sets of 16 images, 8 for condition drop and 8 for condition rise. Each participant saw one of these sets, with alternating drop and rise conditions. That is, for example, an object pair of a ball and a spinning top in condition rise on a green background (see Figure 1a below) was shown to one participant, while another participant saw the same object pair on the same background but in the drop condition. This ensured that object pairs, background, and orientation were counterbalanced across participants.

The project was implemented with open access software. The experiment was built with Python (v. 3.8.). A connection to the Tobii x120 eye tracker was established via the tobii_research software development kit (SDK). For our analyses, we processed data with Pandas (McKinney 2010) and calculated statistical tests in JASP (v. 0.16). Some parts of the code were modified pieces from other projects on open access eye tracking experimental design and analysis (Dalmaijer et al. 2014; Ghose et al. 2020). All scripts, stimuli, and data required to replicate the full experiment and repeat the data analyses are accessible via OSF: https://osf.io/xrbzg/?view_only=13cf79f5e8eb47afab6982fb1731cd12.

### 1.3 Procedure

After passing through our Covid19 protocol (self‐tests for infants and parents), we accompanied participants to our testing room. Infants sat on their parents' lap in front of a screen‐based Tobii x120 eye tracker. Parents were asked to wear mirrored sunglasses so their gaze would not interfere with the eye tracking measurement. We asked parents to keep in the background and not to say anything or point to the screen. The position of the infant was adapted so that their head was centered in front of the screen and the distance to the screen was approximately 60 cm. A five‐point infant‐friendly calibration was conducted to ensure accurate gaze localization. Each trial was individually started by the experimenter. The experiment was paused if the infant was distracted. The measurement continued until all 16 trials had been played or until the infants lost interest in the screen. No trial was shown twice.

### 1.4 Paradigm

The experiment had a baseline, contingent, and disruption phase (Figure 1). In the baseline phase, infants' spontaneous looking behavior to an image stimulus was recorded. For 5 s, infants were able to explore the stimulus and learn the location of the objects. Before the contingent phase, the image was covered with a unicolored surface in a dynamic animation. Small blue or pink squares flew across the screen for 3 s, progressively covering the image. Once the transition was complete, the contingent phase began (Figure 1b). From this moment on, wherever the eye tracker detected a gaze point, a small square of the cover disappeared, and the image beneath was uncovered. This contingency led to the scratching effect that, by gazing over the screen, the stimulus could be scratched free. This phase lasted until either 20% of the stimulus was uncovered or until 30 s had passed. Immediately after the contingent phase, a disruption phase followed (Figure 1c) where the contingency between gaze point and stimulus presentation was disrupted, and the last still image from the contingent phase was displayed. There was no overt notification signaling the start of the disruption phase. It just happened that the participant was no longer able to scratch free further squares and remained in a merely observational role. Infants gaze on this still image was recorded for 5 s. Thus, one trial had a maximum length of 43 s and could be shorter depending on the size of the uncovered surface in the contingent phase.

### 1.5 Analysis

Infants gaze was recorded with 120 Hz. Fixations were calculated as mean gaze location within 25 pixels for more than 200 ms (following recommendation by Gredebäck et al. 2009). Trial exclusions followed our preregistered criteria: If an infant did not look at the screen for at least 1 s during the baseline phase, the entire trial was excluded. If the infant did not look at the screen for at least 10 s or scratched free at least 20% of the screen during the contingent phase, this trial was excluded from the analysis of the contingent and the disruption phase. If the infant did not look at the screen for at least one fixation during the disruption phase, this trial was excluded from the analysis of the disruption phase.

For each trial and phase, a differential looking score (DLS) was calculated based on the fixation data as follows (cf. Kampis et al. 2021). Four quadrants (AOIs) of the screen containing the objects either in the rise or the drop condition were defined as making up about half of the screen size (55.72%). The DLS was defined as the sum of the looking time to the AOIs containing objects minus the sum of the looking times to the quadrants not containing objects divided by the total looking time to the screen. That is, DLS values were distributed on a spectrum from −1 to 1, where the value 1 indicates exclusive looking into the quadrants containing objects, −1 into the quadrants not containing objects, and the value 0 equal looking to all four quadrants. That is, if infants indeed looked more to the AOIs that contained the objects, the DLS should be positive. The DLS of all trials was averaged per condition and subject, and the subject means entered the statistical analyses. Note that the definition of the DLS deviates from the preregistration as it was modified to simplify reporting in response to the suggestion of a reviewer.

The analyses were conducted in a Bayesian sequential framework which allows continuing testing sequentially until sufficient evidence for and against an effect is obtained. Bayesian statistics makes use of model comparisons where prior probability distributions for each model and all parameters involved need to be specified. Having no a priori information about our effects, we used an uninformed default prior, setting equal probabilities for all models (Rouder et al. 2012). Based on the specified prior distributions, each model's marginal likelihood, given the observed data, was calculated and contrasted using the Bayes Factor (BF_10_). The BF_10_ sets the evidence for the alternate model in contrast to the evidence for the null model. The following margins represent a widely accepted classification scheme for the interpretation of the Bayes Factor. A BF_10_ between 1 and 3 is considered anecdotal evidence for the alternate model, between 3 and 10 moderate evidence, greater than 10 strong evidence, greater than 30 very strong evidence, and greater than 100 extreme evidence. Likewise, evidence for the null model is considered anecdotal for a BF_10_ between 1 and 1/3, moderate between 1/3 and 1/10, strong when smaller than 1/10, very strong when smaller than 1/30, and extreme for a BF_10_ smaller than 1/100. All Bayesian analyses were conducted using JASP (Version 0.16).

Our data collection followed a preregistered sequential testing approach, that is, data collection continued until the BF_10_ of the main hypothesis (that infants would look more to the drop AOIs in the drop condition and more to the rise AOIs in the rise condition during the contingent phase) passed 3 or 1/3. A minimum sample size of *N* = 30 was preregistered based on power simulations using the R packages ‘Superpower’ version 0.1.0 [30] and ‘BayesFactor’ version 0.9.12‐4.2 (Morey et al. 2015) following recommendation by Schönbrodt and Wagenmakers (2018). As preregistered, we used non‐parametric equivalents to the preregistered tests whenever feasible as q‐q‐plots indicated a non‐normal distribution of the subject means.

## 2 Results

**FIGURE 2:**
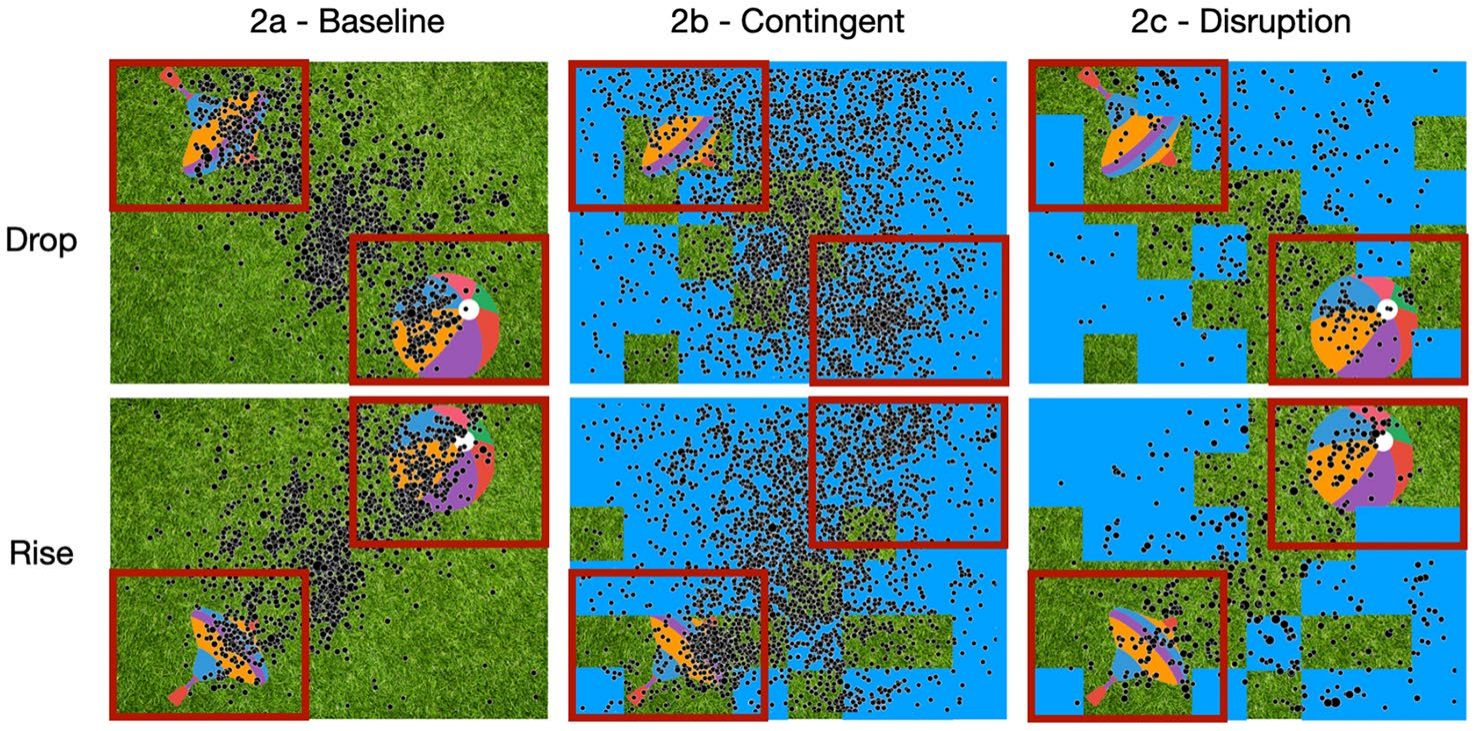
Fixation distribution across all infants plotted on an illustrative image for the baseline, contingent, and disruption phase from left to right. Image condition drop depicted in the top row and image condition rise in the bottom row. AOIs for object locations are illustrated with red squares. For all three phases fixations are visibly distributed along the diagonal axis on which both objects are located.

**FIGURE 3:**
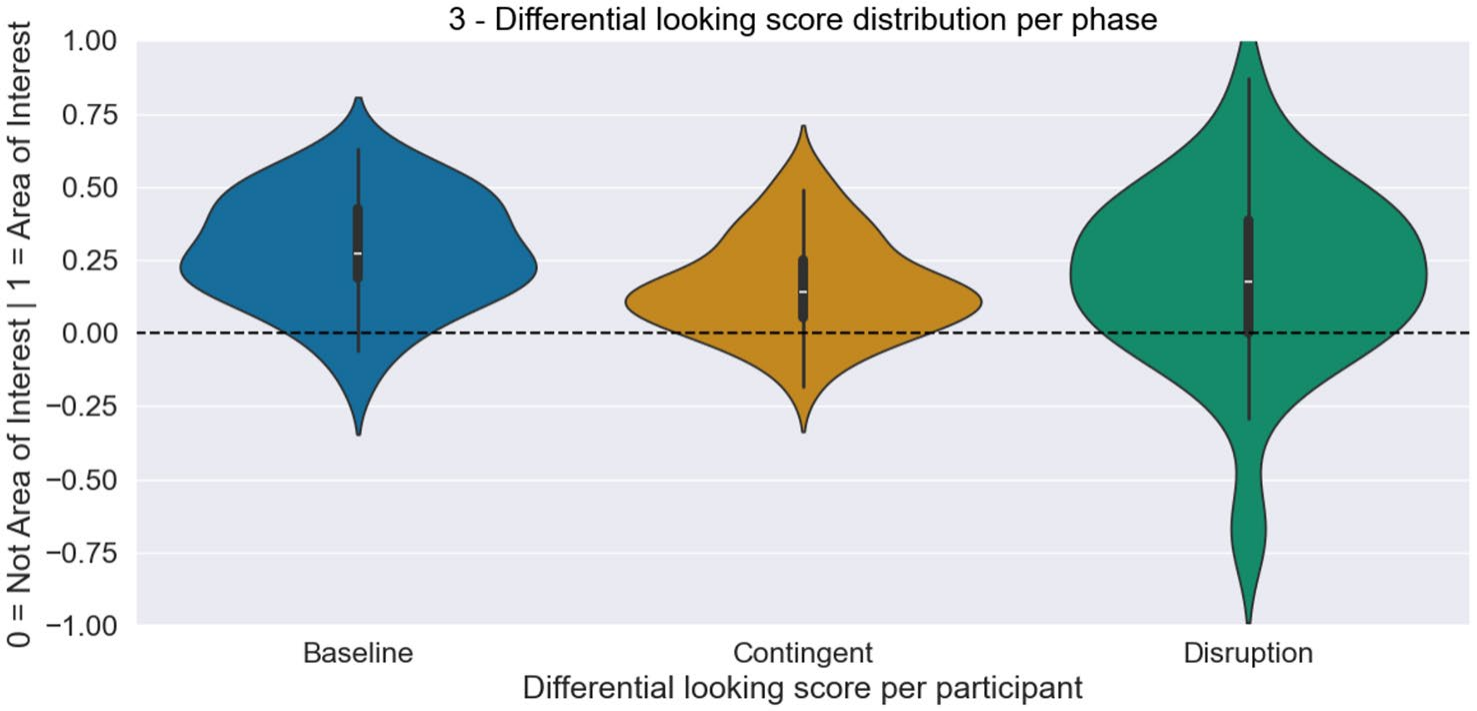
Distribution of the DLS (reflecting looking to areas of the image that contained objects compared to areas that did not) for the baseline, contingent, and disruption phase (from left to right). Distributions are overall skewed toward positive values, indicating that infants looked more toward the areas containing objects in all three phases.

As expected, infants fixated on both objects in the baseline phase (Figure 2a). For the baseline phase, a directed one‐sample Bayesian Wilcoxon Signed‐Rank test yielded extreme evidence for a DLS greater than 0 (*n* = 45, *M* = 0.284, SD = 0.189, SE = 0.028, BF_10_ = 127,863.098, Rhat = 1.026; Figure 3). Similarly, in the contingent phase, the fixation pattern was most dense over the diagonal connecting the AOIs (Figure 2b). More fixations were observed compared to the baseline phase due to the longer duration of this phase. For the contingent phase as well, a directed one‐sample Bayesian Wilcoxon Signed‐Rank test yielded extreme evidence for a DLS greater than 0 (*n* = 45, *M* = 0.194, SD = 0.165, SE = 0.025, BF_10_ = 250603.179, Rhat = 1.049; Figure 3). In the disruption phase, the gaze pattern was again similar with more fixations on the diagonal line between the objects (Figure 2c). A directed one‐sample Bayesian Wilcoxon Signed‐Rank test again yielded extreme evidence for a DLS greater than 0 (*n* = 45, *M* = 0.194, SD = 0.307, SE = 0.046, BF_10_ = 694.333, Rhat = 1.006; Figure 3). A repeated‐measures ANOVA showed a difference between the DLS of the three phases (BF_10_ = 3.438). Post hoc paired‐sample Bayesian t‐test comparisons revealed that this effect was driven by a difference between the baseline and the contingent phase (BF_10_ = 617.99), whereas there was evidence against a difference between the DLS in the contingent and the disruption phase (BF_10_ = 0.204) and the baseline and disruption phase (BF_10_ = 0.824).

**FIGURE 4:**
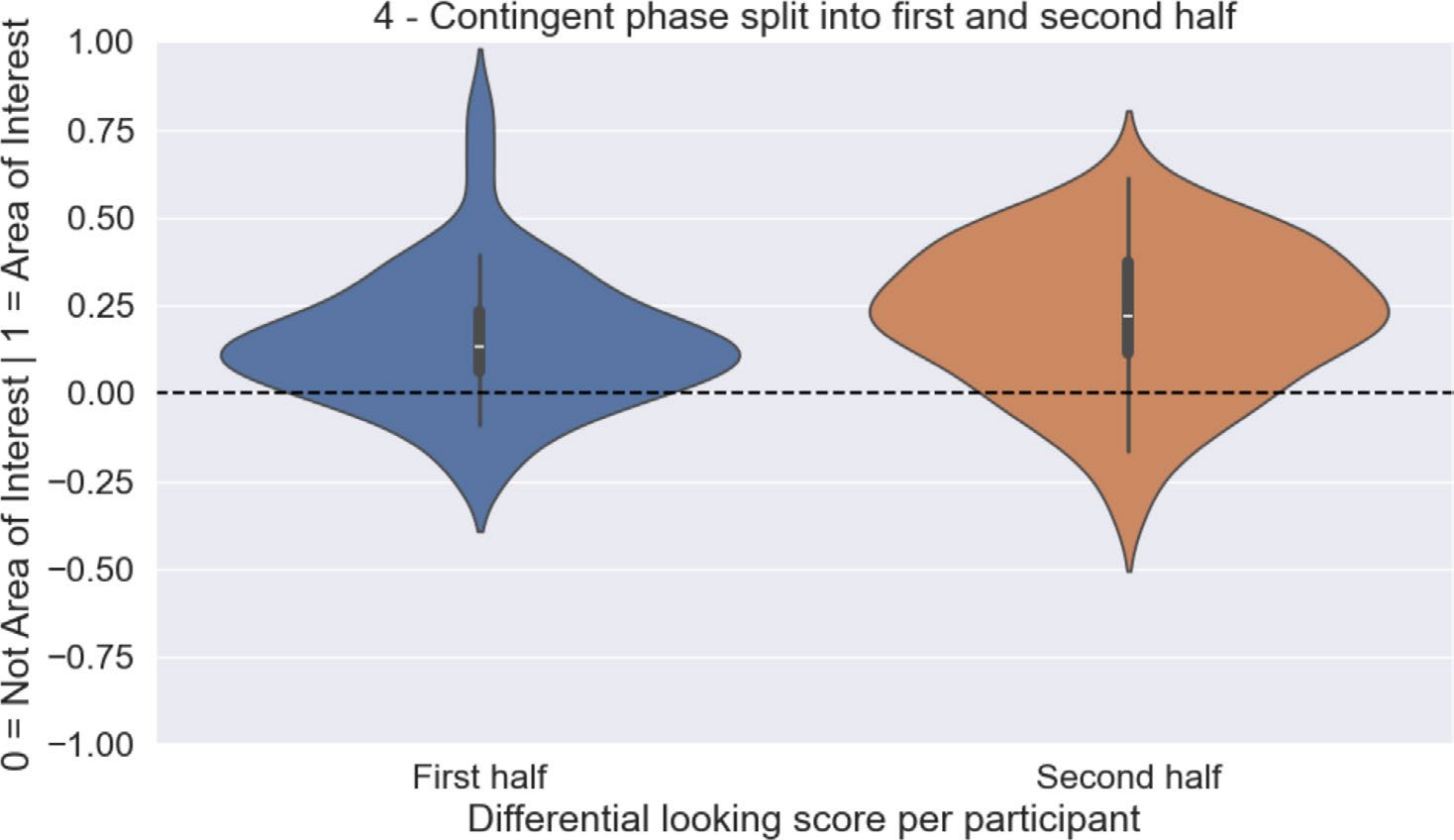
Distribution of the DLS to the areas of interest split into first and second halves of the contingent phase. Infants looked longer at the areas containing objects in the second half than in the first half of the contingent phase.

In the contingent phase, infants could uncover the image by directing their gaze over the covered surface on the screen. The DLS analysis shows that they preferentially directed their gaze toward the regions on the screen that contained objects. This indicates that infants learned about the contingency and used it to uncover the objects. An alternative explanation could have been that infants were biased toward the areas depicting objects in the baseline phase. To exclude this, we conducted a follow‐up analysis where we divided the fixation data from the contingent phase into the first and second half of the phase. A paired‐sample Bayesian Wilcoxon Signed‐Rank test yielded strong evidence that the DLS in the first half of the contingent phase was smaller than the DLS of the second half (n45, *M*_first_ = 0.15, SD_first_ = 0.191, SE_first_ = 0.029, *M*_second_ = 0.216, SD_second_ = 0.206, SE_second_ = 0.031, BF_10_ = 22.05, Rhat = 1.006; Figure 4). This shows that infants looked longer toward the area of interest in the second half of the contingent phase than in the first half, indicating that they directed their gaze more to the objects as they learned about the contingency. This pattern cannot be explained by an attentional bias from the baseline phase, as such a bias would have resulted in strongest looking to object areas in the beginning of the trial.

**FIGURE 5:**
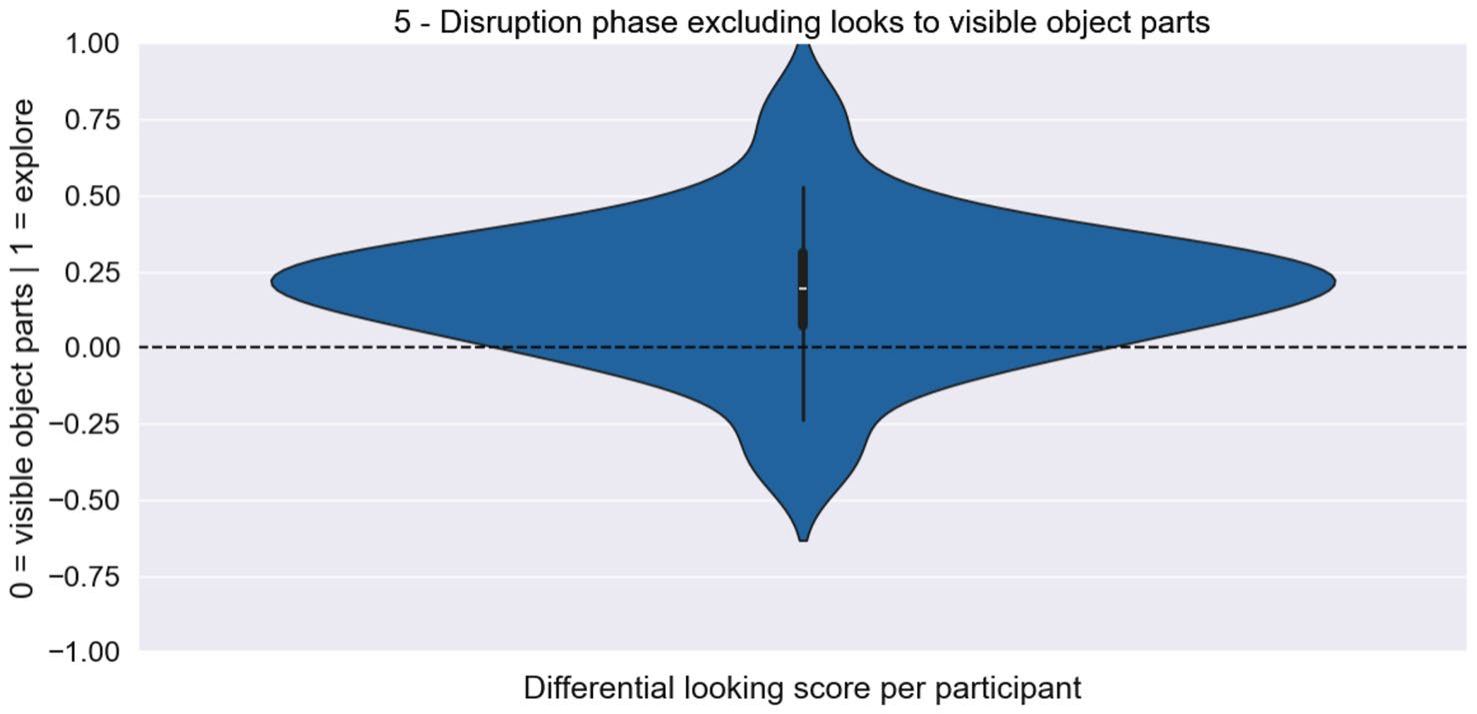
Distribution of the DLS for the disruption phase when fixations to already uncovered object parts were excluded. The DLS was calculated as defined above, except that fixations to visible object parts were subtracted from the looking duration to the quadrants containing objects. This modified DLS was greater than 0, indicating that infants indeed tried to scratch free further parts of the object and that their gaze was not merely attracted by already visible object parts.

In the disruption phase, infants could explore the image that resulted from their scratching movements in the contingent phase. Thus, the images may have contained parts of the objects that had already been scratched free, which could simply have attracted infants' gaze and led to the observed gaze pattern, instead of controlled gaze shifts to uncover the underlying image. To exclude this possibility, we ran an additional exploratory analysis where we excluded fixations to parts of the object that had already been scratched free (referred to as fixations to visible object parts) and only considered fixations to still covered areas of the image (referred to as ‘exploratory’ fixations). That is, we calculated the DLS as before but excluding fixations to already uncovered object parts from the looking duration to the quadrants containing objects., A one‐sample Bayesian Wilcoxon Signed‐Rank test yielded extreme evidence that this modified DLS was also greater than 0 (*n* = 45, *M*_explore_ = 0.188, SD_explore_ = 0.232, SE_explore_ = 0.035, BF_10_ = 27047.879, Rhat = 1.004; Figure 5). This shows that the effect was not driven by infants' gaze toward visible object parts, but by exploratory looks to the unicolored areas still covering the objects. This suggests that infants indeed tried to scratch free further parts of the objects, although the contingency had been interrupted.

**FIGURE 6:**
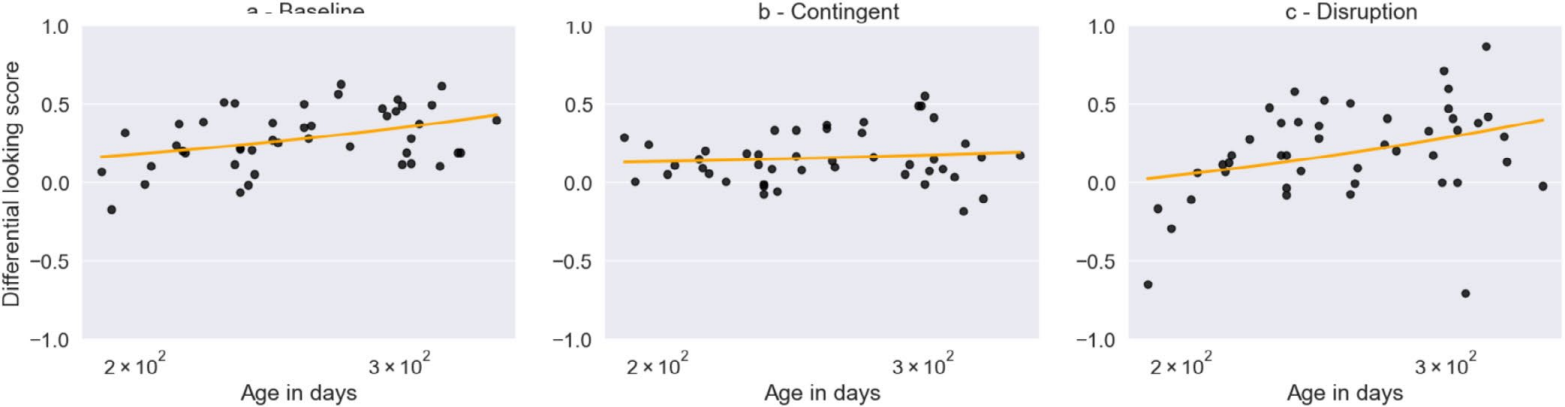
Correlation of age with the DLS for the Baseline (a), Contingent (b) and Disruption phase (c).

To test whether infants' targeted looks to the AOIs increased with age, we correlated infants' age (in days) with the DLS for all three phases separately. This yielded moderate evidence for a positive correlation of infants' DLS with age in the baseline phase (Kendal's tauB = 0.235, BF_10_ = 4.801; Figure 6a), inconclusive evidence in the contingent phase (Kendal's tauB = 0.061, BF_10_ = 0.330; Figure 6b), and strong evidence for a positive correlation with age in the disruption phase (Kendal's tauB = 0.269, BF_10_ = 10.489; Figure 6c).

## 3 Discussion

The ability to control one's movement is a core characteristic of agency. This is what differentiates what merely happens to a person from what they make happen. A marker of agentic control is the ability to flexibly adapt one's actions to changing circumstances. To investigate whether infants possess such control and how agentic control develops between 6 and 10 months, we tested infants' ability to flexibly control their eye movements in an eye tracking experiment where they were able to scratch free different images underlying a unicolored surface with their gaze. We found that infants flexibly adapted their eye movements depending on where they could reveal most information from the underlying image.

Infants were first presented with the images in a baseline phase, which established that infants indeed looked more at objects depicted in some corners of the image than at the background. In the following contingent phase, infants could scratch free the images with their gaze. Despite no longer seeing the objects, infants looked more at the quadrants containing objects in the underlying image than at the quadrants without an object. They flexibly adapted this gaze behavior on a trial‐by‐trial basis depending on where the object could be found in the underlying image. Importantly, this effect was found although the image was fully covered at the beginning of the contingent phase. Further, the effect was greater in the second half of the contingent phase than in the first half. That is, infants' looks at the objects increased throughout the contingent phase. This indicates that, as infants learned about the contingency, they increasingly directed their gaze to uncover the objects on the screen. Even after the contingency between infants' gaze and the scratching of the screen was interrupted in the disruption phase, infants continued to gaze more at the areas containing objects in the underlying image. That is, infants flexibly controlled their gaze to uncover information, even when this behavior was no longer positively reinforced. This enhanced gazing at the object areas increased with age between 6 and 10 months.

In the disruption phase, infants saw the outcome of their scratching activity from the contingent phase as a still image and were no longer able to scratch free more of the image. This phase intended to test whether infants would continue to try scratching free the most interesting areas of the underlying image, although this behavior was no longer positively reinforced. However, depending on the infants' previous scratching activity, parts of the objects were already visible on the screen. To rule out the alternative explanation that infants' looking pattern in the disruption phase may have been driven by the saliency of already visible object parts, we repeated the analysis while excluding looks to already visible object parts. This showed that infants' differential looking to the object areas was indeed driven by their exploratory looks to still covered object areas rather than looks to visible object parts. That is, infants did not merely gaze at attractive salient parts of the image but directed their gaze to yet‐to‐be uncovered pixels. This indicates that they flexibly adapted their eye movements to the information structure of the underlying image in an attempt to uncover the most interesting parts of the image. Thus, infants appear to have understood that they had the possibility to scratch free the image with their gaze and controlled their eye movements in order to do so.

Infants' flexible adaptation of their gaze to areas where more information could be revealed both in the contingent and the disruption phase confirms that infants (a) learned the contingency, (b) remembered the location of the objects, and critically, (c) were able to direct their gaze in a flexible way to where they expected to be able to reveal the objects. That is, infants in this sample learned about the contingency between where they directed their gaze and what happened on the screen, and then, based on this knowledge, they flexibly controlled their gaze to search for the target objects. This interpretation is supported by observations made in the reported sample (*N* = 45, 18 female, recruited in a city of about 600.000 inhabitants in Germany in 2022). Whether it is generalizable to a broader population should be determined through follow‐up studies and replication attempts.

The central findings in our work are based on infants' expectations about where to find information. Previous studies have shown that infants from 6 to 7 months are able to allocate spatial attention and show anticipatory looks following immediately preceding visual cues to the location of interest (Posner 1980; Sheese et al. 2008). In our paradigm, no such immediate visual cue to a location was given. Instead, infants first had to learn about a contingency that occurred on the whole screen. They then had to remember the underlying image and could only then gaze at the areas where more informative content could be uncovered. Indeed, infants' looking at the target areas increased over the course of the contingent phase. This observed progression excludes an alternative explanation according to which infants would merely have continued gazing at the objects because their attention was biased to these locations from the previous baseline phase. Not only did infants have an expectation about where information would be on the screen, but they had to connect this expectation with their control over the contingency to uncover the information, in the absence of a visual cue to it. Our paradigm extends previous studies on agency in several important ways. It extends previous contingency paradigms by including a specific measure of infants' control and flexible adaptation of their movements to changing circumstances. Flexible control is a critical marker of agency. It allows us to distinguish movements that merely happen to an infant (e.g., externally elicited movements) from movements that are initiated and controlled by the infant. Hence, our experiment resolves a critical point that has been raised as a shortcoming of other contingency paradigms like the mobile paradigm (Bednarski et al. 2022). Infants' clear preference for scratching free the changing object locations both in the contingent and the disruption phase shows that their eye movements to unicolored areas of the screen were not merely elicited by the external reinforcement of a change happening on the screen. Instead, infants adapted their gaze in a controlled and flexible way based on the expected information content. Further, they continued to do so after no longer finding more information due to the disruption of the contingency, suggesting that they probed the contingency after it was interrupted in the disruption phase. Importantly, if infants were merely enjoying the contingency between their gaze and the scratching effect, their fixations should not have varied depending on the underlying image. Instead, infants reliably directed their gaze to the corners of the screen where objects could be uncovered. Importantly, these were not always the same corners, but they adjusted their scratching depending on the underlying image. Thus, infants flexibly controlled their gaze based on their goal of uncovering the objects and not merely in response to the rewarding scratching effect.

In contrast to most previous paradigms investigating infants' control of movements (Gottwald 2018; von Hofsten 2004), we built on infants' eye movements. Control over eye movement emerges earlier than control over limb movement (Oakes 2023), which makes vision the ideal modality to test early displays of agency in infancy. Further, this allowed us to measure gradual improvements in control from early on in infancy, and thus to target the developmental trajectory of agency. Indeed, infants' differential looking to the areas containing the objects compared to other areas increased between 6 and 10 months of age. This was the case when the objects were visible in the baseline phase as well as for still covered objects in the disruption phase. For the baseline phase, this reflects infants increasing interest in the objects. In the disruption phase, the positive correlation with age could reflect several factors: First, infants' ability to remember and predict where the objects are located might improve. Secondly, the correlation with age may reflect their increasing ability to exert control over their eye movements in order to direct them to the changing target locations. The latter especially would speak for a development of flexible control in the tested age range. Thus, while our paradigm reveals signs of agency by 6 months, agentic control seems to further develop between 6 and 10 months. Exciting avenues for future research would be to investigate the emergence of agentic control before 6 months and its further development beyond the first year of life.

Miyazaki et al. (2014) provided initial evidence for infants' ability to learn a contingency between their gaze and a visual stimulus. Our results concur with these findings and at the same time significantly extend their work. With our baseline phase, we provided infants with prior information, which they could use to update their predictions about where to find interesting information. By systematically varying where in the image more information could be found, we were able to measure infants' flexible adaptation of their gaze dependent on the information content. In contrast to theoretical work by Rochat (2007) and Kelso (2016), our study emphasizes the importance of measuring flexible control when studying the mental processes involved in agency.

Another relevant mental process involved in agency is the agent's perception of themselves as being in control, often referred to as a beings ‘Sense of Agency’ (Carruthers 2012; De Haan and de Bruin 2010; Haggard 2005). In the present research, we do not capture this aspect but instead focus on infants' ability to exert control. This is because we argue that it is not possible to test how non‐verbal infants perceive a situation (Bednarski et al. 2022). One possibility to get closer to testing for a sense of agency could be to extend our gaze‐contingent paradigm to include degrees of difficulty in detecting agency within the scratching phase, for example by varying the size of the area that is uncovered by participants' gaze point. Systematically differing behaviors in response to different degrees of difficulty could potentially reveal how strongly participants experience their own agency, which would need to be established in adults (who are able to report their experience) first. Nevertheless, it cannot be concluded that what looks like similar gaze behaviors between infants and adults on the surface, corresponds to the same underlying experience.

Another interesting extension would be to manipulate the value of the information contained in each image, for example, by varying its saliency, meaning, or normative content (Kanakogi et al. 2022; Oakes et al. 2024). Previous research (Kenward 2010; Klossek et al. 2008) using outcome devaluation paradigms showed that infants from about 2 years of age are sensitive to the value of a stimulus for them. Klossek et al., for example, showed that older infants (> 2.5 years) performed a trained behavior less often than younger infants (< 2 years) when the reward for that behavior was decreased. Further, Kanakogi et al. show that 8‐month‐old infants direct their gaze to an aggressive agent if they have previously learned that their gaze causes the agent to be punished. This indicates that infants can direct their gaze toward a visible target to produce a previously learned contingency (i.e., the punishment). Similar adaptations to the paradigm presented here could be used in the future to measure degrees to which infants' control is influenced by the information to be revealed in the contingent phase. The information could be devalued from trial to trial by repeating itself (e.g., as in Klossek et al. 2008) or the information could be normatively loaded by including images of social or antisocial agents (e.g., as in Kanakogi et al. 2022). It is worth noting that the value as perceived by an infant participant possibly influenced the degree of control exerted but not the possession of control itself. Our current research only shows possession and exertion of control across a group of infants and not individual degrees of control dependent on internal psychological states. Degrees of control could further be analyzed by examination of the temporal sequence of scratching patterns across the screen. This could reveal the ways in which infants begin to examine the contingency and learn to uncover information throughout the contingent phase.

Being in control over interactions with the environment allows infants to create and shape their own learning opportunities, and thus fosters learning and further development. Infants who possess the ability to flexibly control their eye movements can determine by themselves which aspects of their environment they want to receive visual information. It is no longer merely external stimulation from the environment that forces information upon them, but their own controlled action that allows them to guide their field of view to information of interest to them. In other words, through the development of agency, infants learn that they can explore the world by themselves. Indeed, research on information seeking (Altmann et al. 2023; Poli et al. 2020, 2024) has shown that infants build predictions about where to expect information and by updating those predictions they are able to maximize their learning progress. An interesting question for future research is how the development of flexible control fosters infants' information seeking behavior and their improved capacity to learn from their environment.

## 4 Conclusion

In sum, our paradigm demonstrates how control defined as a subject's ability to flexibly adapt bodily motion to changing circumstances can be used as a marker of agency in behavioral observations. We show that, between 6 and 10 months of age, infants have significant abilities to exert control over their gaze, which they use to direct it to changing locations of anticipated richer information. This flexible adaptation of their gaze increases between 6 and 10 months, suggesting that by 6 months of age infants can be considered agents, and that agentic control further develops in the first year of life. The development of this ability is a significant milestone on infants' path to becoming autonomous agents.

## Author Contributions

**Florian Markus Bednarski:** conceptualization, methodology, software, formal analysis, and writing – original draft. **Katrin Rothmaler:** methodology, formal analysis, writing – review and editing, and supervision. **Simon M. Hofmann:** methodology, software, writing – review and editing. **Charlotte Grosse Wiesmann:** conceptualization, methodology, writing – review and editing, and supervision.

## Supporting information


Data S1.

## Data Availability

All resources required to replicate this experiment can be accessed online. Data are available at the following URL: https://osf.io/xrbzg/?view_only=13cf79f5e8eb47afab6982fb1731cd12. The analytic code necessary to reproduce the analyses presented in this paper is publicly accessible. Code is available at the following URL: https://github.com/florianbednarski/Gaze‐Scratch‐Paradigm. The materials necessary to attempt to replicate the findings presented here are publicly accessible. Materials are available at the following URL: https://osf.io/xrbzg/?view_only=d850802eaf404708b4e81660e88dff41. The main analyses presented here were preregistered. The preregistration is available at the following URL: https://aspredicted.org/kvjs‐bxv5.pdf.
