# Supplementary material for: Infants in Control—Evidence for Agency in 6‐ to 10‐Months‐Old Infants in a Gaze‐Contingent Eye Tracking Paradigm
Source: Child Dev. 2025 Jul 25;96(6):1968–77. doi: 10.1111/cdev.70022 (PMC12598448; doi:10.1111/cdev.70022)
Supplement: Supplementary file 1 — Data S1. [file CDEV-96-1968-s001.docx]

SUPPLEMENTARY MATERIALS

Summary of the pre-registered analyses and descriptive data inspections. Note that the calculation of the differential looking score was changed in response to a reviewer request. These supplementary materials demonstrate the Bayesian Sequential Analysis approach.

N= 45 Bayesian Sequential Analysis Plots for BF10 of Contingent Phase DLS difference (drop/rise):


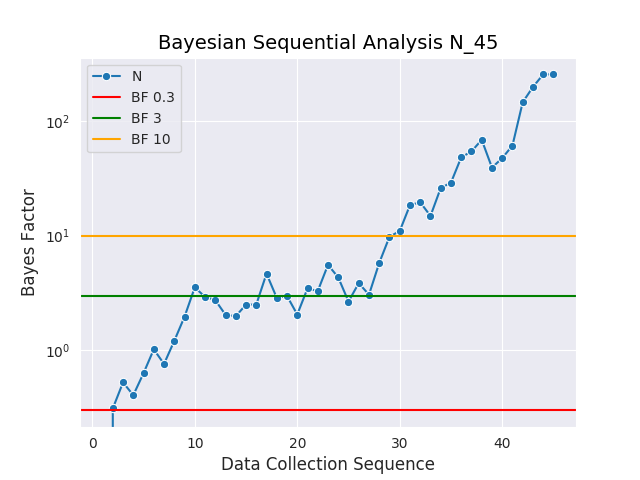


N= 30 Bayesian Sequential Analysis Plots for BF10 of Contingent Phase DLS difference (drop/rise):


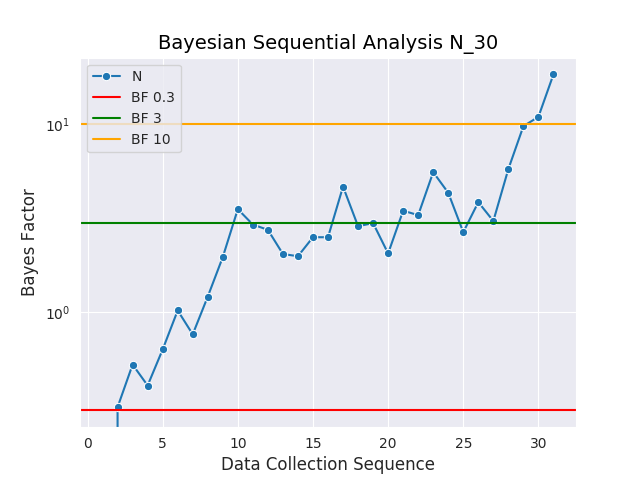


N=30 pre-registered Analysis for DLS difference with Bayesian Wilcoxon Signed-Rank test and Q Q Plots for Baseline, Contingent, Disruption Phase nominal Difference in DLS:

Baseline

For the pre-registered minimum sample size of N = 30, a directed paired Bayesian Wilcoxon Signed-Rank test provided extreme evidence for a higher DLS for images of the condition rise than for images of the condition drop (n=30, M_rise_ = 0.693, SD_rise_ = 0.583, SE_rise_ = 0.106, M_drop_ = -0.644, SD_drop_ = 0.599, SE_drop_ = 0.109, BF = 29328.235, Rhat = 1.036).

Two directed one sample Bayesian Wilcoxon Signed-Rank test confirmed the tendency to look at the quadrants containing objects for the drop and the rise condition (BF_drop_ = 3647.561, Rhat_drop_ = 1.001, BF_rise_ = 855.124, Rhat_rise_ = 1.006).


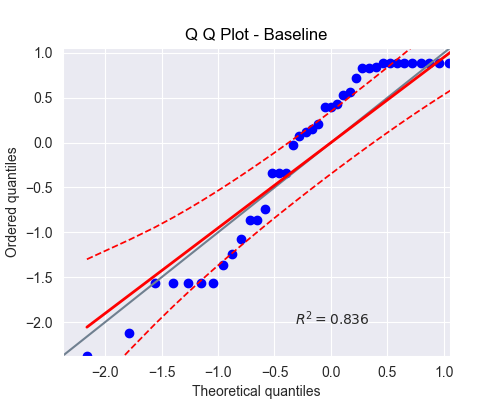


Contingent

For the pre-registered minimum sample size of N = 30, a directed paired Bayesian Wilcoxon Signed-Rank test provided strong evidence for a higher DLS for images of the condition rise than for images of the condition drop (n=30, M_rise_ = 0.044, SD_rise_ = 0.561, SE_rise_ = 0.103, M_drop_ = -0.170, SD_drop_ = 0.605, SE_drop_ = 0.110, BF = 10.891, Rhat = 1.001).

Two directed one sample Bayesian Wilcoxon Signed-Rank test confirmed the tendency to look at the quadrants containing objects for the drop and the rise condition (BF_drop_ = 1.015, Rhat_drop_ = 1.001, BF_rise_ = 0.233, Rhat_rise_ = 1.000).


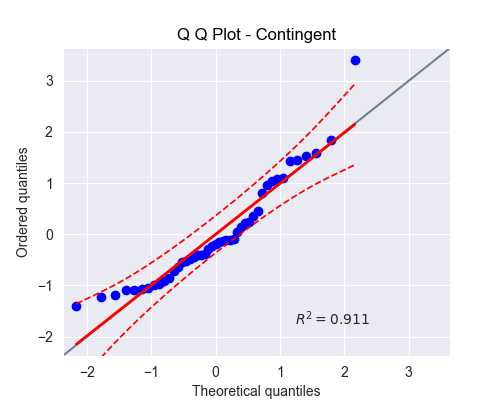


Disruption

For the pre-registered minimum sample size of N = 30, a directed paired Bayesian Wilcoxon Signed-Rank test provided extreme evidence for a higher DLS for images of the condition rise than for images of the condition drop (n=30, M_rise_ = 0.164, SD_rise_ = 0.604, SE_rise_ = 0.110, M_drop_ = -0.351, SD_drop_ = 0.632, SE_drop_ = 0.115, BF = 414.158, Rhat = 1.001).

Two directed one sample Bayesian Wilcoxon Signed-Rank test confirmed the tendency to look at the quadrants containing objects for the drop and the rise condition (BF_drop_ = 17.603, Rhat_drop_ = 1.001, BF_rise_ = 1.048, Rhat_rise_ = 1.000).


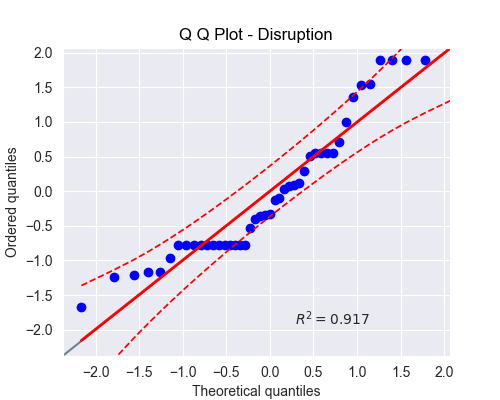


Exclusion rate of trial for n45 infants per phase of the paradigm:

Overall, these 45 infants contributed a total of 1955 recorded trials, 465 of these were excluded (exclusion rate of 24%) according to criteria stated in the preregistration (i.e., insufficient looking time to the screen). For the 6 months-old infants the exclusion rate was 20%, for 7 months-old 21%, for 8 months-old 23%, for 9 months-old 30% and for 10 months-old 29%.


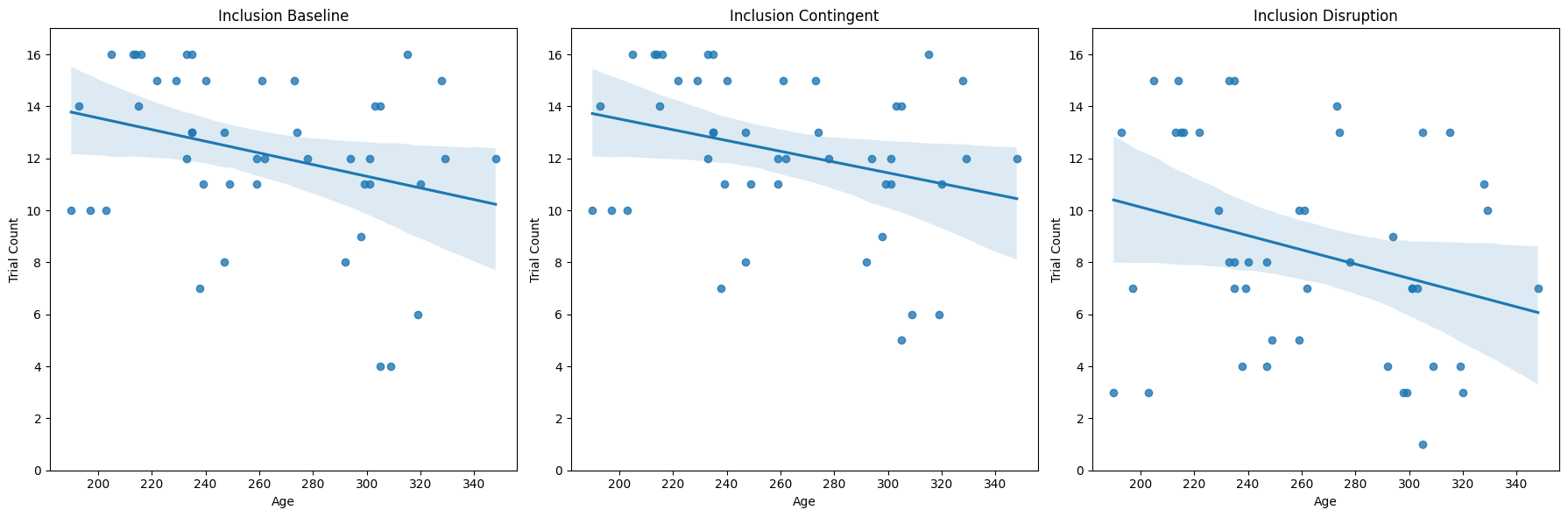


From left to right, blue scatter and line plot represent included trials per age. The maximum number of trials per participant in this study was 16.
